# Effects of Dietary Pork Fat Cooked Using Different Methods on Glucose and Lipid Metabolism, Liver Inflammation and Gut Microbiota in Rats

**DOI:** 10.3390/foods10123030

**Published:** 2021-12-06

**Authors:** Wenzheng Zhu, Yan Xu, Jun Liu, Dawei Chen, Huimin Zhang, Zhangping Yang, Xiaoyan Zhou

**Affiliations:** 1 Engineering Research Center for Huaiyang Cuisin of Jiangsu Province, College of Tourism and Culinary, Yangzhou University, Yangzhou 225127, China; zhuwz@yzu.edu.cn (W.Z.); xuyanlp@yzu.edu.cn (Y.X.); yzuxyz@163.com (X.Z.); 2 College of Animal Science and Technology, Yangzhou University, Yangzhou 225009, China; hmzhang@yzu.edu.cn; 3 Key Laboratory of Chinese Cuisine Intangible Cultural Heritage Technology Inheritance, Ministry of Culture and Tourism, Yangzhou University, Yangzhou 225127, China; 4 College of Food Science and Engineering, Yangzhou University, Yangzhou 225127, China; junliu@yzu.edu.cn (J.L.); dwchen@yzu.edu.cn (D.C.)

**Keywords:** cooking method, pork fat, glucose and lipid metabolism, liver inflammation, intestinal microbiota

## Abstract

Cooking may affect the nutritional value of pork fat, and, nowadays, people have been paying an increasing amount of attention to the method of cooking. In this study, the effects of dietary pork fat cooked using different methods on body metabolism and intestinal microbes were studied in rats. Fat was extracted from pork belly meat cooked using three methods: braising (braising cooking method, BCM), stewing (SCM) and deep fat frying (DCM). The three types of pork fat were added to animal feed, and the effects of each on body weight, glucose and lipid metabolism, liver inflammation and intestinal microbes in rats were compared with the effects of soybean oil-treated feed (SO) and a blank control (BC). Rats in all three groups fed with cooked pork fat exhibited significant increases in body weight compared with the controls across the experimental feeding period. Furthermore, all three types of pork fat led to significant changes in the serum concentrations of triglycerides (TG) and total cholesterol (TC) relative to the controls, with the greatest increases in TG and TC in the BCM and DCM groups, respectively. All three types of pork fat led to significant decreases in serum high-density lipoprotein cholesterol concentrations relative to the controls, with the lowest concentration in the SCM group. All three types of pork fat also led to significant increases in low-density lipoprotein cholesterol concentrations relative to the controls, with the smallest increase in the DCM group. Rats in the SCM group had the highest level of liver fat deposition, followed by those in the BCM, DCM, SO and BC groups. Compared with the controls, the three groups fed with different types of cooked pork fat had significantly lower hepatic expression of nuclear transcription factor kappa B (NF-κB). The expression levels of NF-κB in the DCM and SO groups were significantly lower than those in the other groups. The abundance of Proteobacteria species in the intestines of rats was significantly lower in the BC group than in the other groups fed with cooked pork fat, and the abundance of Bacteroidetes species was significantly lower in the BCM, SCM and DCM groups than in the BC and SO groups. From the changes in the abundance of Firmicutes and Bacteroides, pork fat in the three cooking methods has a certain potential to promote the production of body obesity.

## 1. Introduction

In China, one of the largest meat-producing countries in the world, pork is the most frequently consumed type of meat by residents [1]. Pork is cooked using various methods, such as braising, stewing and deep frying, and many recipes prepared using these different methods have become classic dishes. Over time, gradual improvements in lifestyles, nutrition and health awareness have led people to pay attention to healthy cooking methods. China’s National Nutrition Plan (2017–2030) proposes to transform traditional cooking methods to improve the nutritional quality of foods and develop healthy cooking methods that can maximize the nutritional value of foods and meals based on scientific data and thus improve human health.

Cooking causes a series of changes in the physical and chemical properties of food. In pork, cooking at a high temperature causes lipid oxidization, which produces rich flavor substances [2]. The cooking method, temperature and time can accelerate the rate of lipid oxidation, and these changes can produce peculiar aromas, textural changes and toxic compounds and induce rancidity [3]. Additionally, the cooking method and time significantly affect the fatty acid composition, fat content and nutrition and flavor profiles of pork, and the processing method has a particular effect on fat content. Li et al. [4] found that cooking increases the protein and polyunsaturated fatty acid (PUFA) content and decreases the fat content of pork belly. Rasinska et al. [5] studied the effects of different cooking methods on lipid oxidation in rabbit meat and found that roasting increased lipid oxidation to a greater extent than boiling. Lyulin et al. [6] studied the effects of different cooking methods, such as boiling, baking and frying, on lipid oxidation in fish fillets and found that baking and frying more readily led to increases in the carbonyl group and Schiff base contents and reduced the free sulphur content. Zhang et al. [7] studied the effects of cooking methods, such as steaming, boiling, frying and roasting, on pork and found that the crude protein and PUFA content increased significantly after cooking.

Pork contains a large amount of saturated fatty acids (SFAs). In humans, an increase in the proportion of SFAs in the body is considered a precursor of hypertension and hyperlipidemia [8], and excessive intake of pork fat is thought to lead to cardiovascular and cerebrovascular diseases. Studies have shown that consuming saturated fatty acid (SFAs) increases the serum cholesterol concentration, while consuming PUFAs reduces the serum cholesterol concentration, particularly that of low-density lipoprotein cholesterol, which is associated with arteriosclerosis [9]. In one study, the level of dietary palmitic acid, an SFA, was shown to be closely associated with the serum concentration of low-density lipoprotein cholesterol (LDL-C) [10]. In nutritional evaluations, therefore, the PUFA/SFA ratio is generally used to evaluate the nutritional value of pork fat [11], such that the higher the PUFA/SFA ratio, the higher nutritional value of the meat.

The cooking method not only affects the nutritional quality of pork but also affects the health of the intestinal microbiota of the organism that consumes the cooked meat. The gut microbiota plays a vital role in glucose and lipid metabolism [12]. Pork fat can slow the passage of food through the stomach and intestines, thereby improving the digestibility of nutrients, such as fat and protein, and contributing to the intestinal microecological balance [13]. Intestinal microbes are affected differently by different fatty acids and by many other factors, such as genetics, geographic origin, age, drugs and diet [14]. Of these, the diet is the main regulator of the composition and function of the intestinal microbiota, such that host–microbe interactions depend on the consumption of appropriate types of food in appropriate forms [15]. As such, dietary fat has an important effect on the gut microbiota. Many studies have shown that intake of a high-fat diet induces changes in the gut microbiota at the phylum and genus levels that lead to obesity and metabolic disorders [16]. Much recent research on dietary fat has focused on disturbances or imbalances of the intestinal microflora caused by high-fat diets, including the effects of pork fat subjected to different processing conditions [17,18]. Although many microbiome studies have compared diets containing different ingredients, there remains a lack of research on the effects of cooking methods [15], even though cooking is a unique, ubiquitous and ancient human practice [19]. This article explores the effects of pork fat cooked using different methods on the structure and function of the gut microbiota in rats.

This article focuses on traditional Chinese dishes that use pork belly as the base ingredient. The meat was cooked using three different methods, namely, braising, stewing and deep frying. The resulting braised pork, stewed pork and deep-fried pork meatballs were treated as our research objects. Fat was extracted from samples of meat cooked using these three methods and fed to rats during their growth period. Accordingly, the rats were categorized according to the consumption of fat from meat cooked using the braised cooking method (BCM), stewed cooking method (SCM) or deep frying cooking method (DCM); other rats were fed a diet supplemented with soybean oil (SO) or designated as blank controls (BC). Changes in the body mass, glucose and lipid metabolism, liver inflammation and intestinal microflora were compared between the groups of rats.

## 2. Materials and Methods

### 2.1. Materials and Equipment

Pork belly, produced by a ternary hybrid pig breed, was purchased from a local supermarket in Yangzhou city in China. Soybean oil was purchased from Yihai Kerry Investment Co., Ltd., Shanghai, China. Trichloromethane, methanol, benzene, petroleum ether, potassium hydroxide and n-hexane were purchased from Sinopharm Chemical Reagent Co., Ltd., Shanghai, China. GLU kit, TC kit, TG kit, LDL-C kit and HDL-C kit were purchased from Meikang Biological Technology Co., Ltd., Ningbo, China. C21S-C2170 Joyoung Induction Cooker: Joyoung Co., Ltd., Hangzhou, China. Temperature-controlled fryer/electric fryer: Hanke Machinery Technology Co., Ltd., Weifang, China. DSQ II gas chromatography-mass spectrometer: Thermo Co., Ltd., Waltham, MA, USA. The 7020 Hitachi automatic biochemical analyzer: Hitachi, Tokyo, Japan. SorvallST16R high-speed refrigerated centrifuge: Thermo Fisher Scientific Co., Ltd., Waltham, MA, USA.

### 2.2. Conditions and Processes of the Three Cooking Methods

According to the literature [20,21] and a preliminary study, the following conditions and processes were set for the three cooking methods.

(1) BCM: Five hundred grams of pork belly was cooked in 3500 mL of water at 100 °C for 15 min and then cut into 2 × 3 × 3 cm blocks, which were sautéed at 160 °C for 5 min without water. Next, 120 g of green onions, 120 g of ginger, 30 g of cooking wine, 2500 mL of water, 20 g of dark soy sauce, 20 g of light soy sauce, 30 g of white sugar and 2 g of salt were added to the pork, which was cooked at 200 W for 90 min in an induction cooker. The obtained samples were collected.
(2) SCM: Five hundred grams of pork belly was cut into 0.6 × 0.5 × 0.5 cm granules and mixed with 6 g of green onions, 6 g of ginger, 8 g of salt and 10 g of starch. The pork pieces were boiled in 2500 mL of water at 100 °C for 3 min, and then the cooked samples were transferred to a saucepan with 2500 mL of water. After stewing at 200 W for 120 min, samples were collected.
(3) DCM: Five hundred grams of pork belly was finely pureed and mixed with 6 g of green onions, 6 g of ginger, 3 g of salt and 10 g of starch. From this mixture, pork balls with a diameter of 2.0 cm (30 g/sample) were made by hand and fried in 2500 mL of soybean oil at 150 °C for 8 min. Samples were collected.

### 2.3. Determination of Fatty Acids

Fatty acid extraction and determination were performed as described in Yu et al. [22], with some modifications, as follows:

(1) For fat extraction, 5 g of pork sample obtained from meat samples cooked using each of the three methods was placed in a Petri dish and dried at 102 °C for 1 h in an oven. The dried samples were ground. Amounts of 0.5 g of each dry sample and 2 mL of a benzene–petroleum ether solution (1:1) were mixed in a centrifuge tube and reacted for 24 h.
(2) After the extraction, 2 mL of a 0.4 mol/L potassium hydroxide-methanol solution was added to each sample, followed by vortexing for 3 min. Next, 100 μL of an internal standard was added to each sample. After a 30 min incubation, an ultrapure water layer was added, and the upper layer of the solution was collected. Next, a certain amount of anhydrous sodium sulphate was added for use.
(3) A 100 μL aliquot of the collected sample layer was diluted with 1 mL of n-hexane, mixed well and passed through a 0.22 µm membrane filter for injection. The free fatty acids in each sample were analyzed by gas chromatography-mass spectrometry (GC-MS).

### 2.4. Preparation of Animal Feed

All of the pork fat samples were extracted from cooked pork belly meat from the BCM group, SCM group and DCM group and were then used in the customized and purified experimental rat feeds. According to Folch et al. [23], the pork fat samples were homogenized in a 2:1 chloroform–methanol (*v/v*) solution. The homogenate was then filtered to extract pork fat, which was placed under reduced pressure in a vacuum drying box. Each sample was vacuum dried for 48 h at a constant temperature of 40 °C to completely remove the remaining organic solvents. The dried fat samples were vacuum sealed and stored at −20 °C.

The experimental rat feed was designed according to the AIN-93G standard feed. The customized feeds were ordered on commission from Trofi Feed Technology Co., Ltd., Nantong, China The feed was vacuum packed and stored at −20 °C, and it was equilibrated to room temperature in advance before feeding. The dietary composition of the rat feed in the experimental groups is shown in Table 1. The fat addition amount of fat from the BCM, SCM, DCM and SO experimental groups into the standard feed was 70 g/kg.

### 2.5. Animals and Experimental Design

Forty specific pathogen-free male Sprague Dawley rats aged 4–6 weeks (mean body weight: 130.5 ± 20.5 g) were purchased from the Animal Experiment Center of Jiangsu University, Zhenjiang, China (Permit No. SCXK 2018-0012). The animal feeding experiments were conducted at the Animal Laboratory of the School of Food Science and Engineering, Yangzhou University, Yangzhou, China (Permit No. SYXK 2017-0044). The animal experimental protocols were approved by the Animal Ethics Committee of Yangzhou University, China (permit no. SYXK2016-0019). The study was conducted using a randomized experimental design. The rats were divided into five experimental groups: the BCM group, SCM group, DCM group, SO group and BC group. Each group contained eight rats. The total duration of the experiment was 63 days, of which the first 7 days was the adaptive acclimation time. All rats in each group were housed in a single cage under a 12 h/12 h light/dark cycle, a temperature range of 20–25 °C and a relative humidity range of 50–60%. During the experiment, the feed was changed every day to prevent oxidative deterioration of fats in the feed. At the end of the experimental feeding period, the rats were fasted for 12 h before the following experimental samples were collected.

(1) Collection of fecal samples: Fresh feces were collected by the extrusion method and snap frozen in liquid nitrogen. The samples were stored in a freezer at −80 °C until testing.
(2) Collection of serum samples: On days 0 (starting time), 14, 28, 42 and 56 of the experiment, capillary blood samples were collected from the rats via the retroorbital route. The samples were centrifuged for 10 min at 5000 rpm and 4 °C to separate the sera. The serum layer of each sample was removed, placed in a 0.5 mL centrifuge tube and stored at −80 °C until testing.
(3) Rat organ collection: On day 56 of feeding, the rats were killed by spinal dislocation, after which the rats were quickly dissected, and the liver tissues were harvested. After washing in normal saline, the tissues were weighed. Two 1.5 × 1.5 cm sections of tissue were cut from the same part of the large liver lobe, snap frozen in liquid nitrogen and stored at −80 °C until testing.

### 2.6. Determination of Rat Body Mass Index

During the experiment, the rats were monitored closely to observe their food intake and growth. Drinking water was regularly refilled. The residual feed was removed and discarded before feeding every day to avoid oxidative deterioration. The rats were weighed at 14-day intervals during the 56-day feeding period, and their weights were recorded.

### 2.7. Determination of Biochemical Indicators of Glucose and Lipids in Rats

The serum concentrations of glucose, total cholesterol (TC), triglycerides (TG), high-density lipoprotein cholesterol (HDL-C), low-density lipoprotein cholesterol (LDL-C) and other blood lipid biochemical indicators were analyzed using an automatic biochemical analyzer (7020 Hitachi, Tokyo, Japan).

### 2.8. Rat Liver Oil Red O Staining Method

A section of the rat liver tissue was dehydrated using a Leica ASP300S automatic tissue dehydrator. Next, the dehydrated tissue was embedded in paraffin using a HistoCore Arcadia H Leica ASP Lycra embedding machine and sectioned using a Leica NANOCUT automatic semi-thin paraffin microtome. The tissue sections were then dyed with oil red O as follows: Frozen liver tissue slides (at −80 °C) were placed at room temperature. The tissues were soaked with 4% paraformaldehyde A for 1 min, followed by 60% isopropanol for 3 min. The sections were then incubated with oil red O for 15 min, rinsed with 60% isopropanol for 1 s and 75% ethanol for 1 s and washed with distilled water for 3 s. The sections were stained with hematoxylin for 20 s and rinsed with tap water. Finally, the sections were mounted using glycerol gelatin and viewed under an OLYMPUS CX23 microscope for image collection and quantitative analysis of HALO oil red area.

### 2.9. Determination of Inflammatory Factor Expression in Rat Livers

The relative concentrations of nuclear transcription factor (NF-κB), tumour necrosis factor-α (TNF-α), interleukin-1β (IL-β) and interleukin-6 (IL-6) were measured using an ELISA kit (Nanjing Jiancheng Institute of Bioengineering, Nanjing, China). The tests were carried out according to the manufacturer’s instructions. First, the rat liver tissue was taken and homogenized, and then 9 times volume of phosphate buffer saline (PBS, pH = 7.4) was added. After sufficient preparation, the liver tissue was centrifuged at 2500 r/min for 20 min at 6 °C, and the supernatant was collected for subsequent experiments. The wells of the ELISA plate were incubated at 37 °C for 30 min with the prepared sample, with standard and biotin-conjugated antibodies added. After washing, HRP-conjugated streptavidin working solution was added and kept at 37 °C for 30 min. The substrate reagent (protected from light) was added to each reaction well, and the color was developed at 37 °C for 15 min. The reaction was stopped with a stop solution. Finally, a microplate reader was used to measure the absorbance of each well at a wavelength of 450 nm. The concentration of NF-κB, TNF-α, IL-1β and IL-6 was calculated according to the standard curve.

### 2.10. Analysis of the Rat Gut Microbiota Structure

(1) Collection and storage of fecal samples. Feces were collected before the rats were sacrificed on day 56 of the feeding period. The collected feces were immediately placed into labeled sterile test tubes, sealed and stored at −80 °C.
(2) DNA extraction and quality identification of fecal flora. Total DNA was extracted from the fecal samples according to the instructions provided with the EZNA Soil kit. The DNA concentration and purity were tested using a NanoDrop 2000 spectrophotometer, and the DNA extraction quality was tested via PCR amplification of the V3–V4 variable region of bacterial 16S rRNA with the 338F (5′-ACTCCTACGGGAGGCAGCAG-3′) and 806R (5′-GGACTACHVGGGTWTCTAAT-3′) primers. The following amplification procedure was used: pre-denaturation for 3 min at 95 °C; 27 cycles of denaturation for 30 s at 95 °C, annealing for 30 s at 55 °C and extension for 30 s at 72 °C; and a final extension for 10 min at 72 °C. All amplification reactions had a total volume of 20 µL, containing 4 µL of 5FastPfu buffer, 2 µL of 2.5 mM dNTPs, 0.8 µL of each primer (5 µM), 0.4 µL of FastPfu polymerase and 10 ng of DNA template. The PCR products were subjected to 1% agarose gel electrophoresis.
(3) Analysis of 16S rRNA data from rat fecal flora. Rat feces were collected after day 56 of feeding, and the DNA was extracted from faces, purified using the AxyPrep DNA Gel Extraction Kit, eluted with Tris-HCL and detected by 2% agarose electrophoresis using QuantiFluorTM-ST for quantification. The purified amplification products were used to construct a PE2300 library according to the Illumina Miseq platform standard operating procedures. Sequencing was performed on the Illumina Miseq PE300 platform (Meiji Biomedical Technology Co., Ltd., Shanghai, China).

### 2.11. Statistical Analysis

Each experimental group contained 8 replicates. The analytical results are expressed as means ± SD and were compared using Bonferroni’s test. For multiple comparisons, a *p*-value < 0.05 indicates a significant difference. Microsoft Office Excel software was used to process the experimental data. GraphPad Prism 7.0 and SPSS 17.0 were used to create graphs and conduct the statistical analysis.

The liver fat deposition index of each group of rats was calculated through the NM algorithm [24]. The analysis was based on the HALO Area Quantification BF (Indica Labs; Albuquerque, NM, USA) algorithm and a quantitative evaluation of fat droplet staining in oil red O-stained tissues. After the algorithm was debugged, it was applied to analyze each tissue slice, and an objective quantitative analysis was performed.

## 3. Results and Discussion

### 3.1. Analysis of Fatty Acid Composition of Pork Fat Cooked Using Different Methods

In the tested samples of cooked pork fat, changes in fatty acids levels were found to be directly related to nutritional quality. The main fatty acid components corresponding to the BCM, SCM, DCM and SO are shown in Table 2. We found significant differences in the SFA contents between pork fat samples cooked using the three cooking methods (*p* < 0.05). The highest SFA content was observed in SCM pork fat, followed by DCM and BCM pork fat. There was no difference in unsaturated fatty acids (UFAs) between SCM and DCM samples. Compared with DCM and SCM, the UFA content in BCM pork fat was relatively low. In the relevant literature, Gu et al. [25] reported that cooking significantly decreased the proportion of SFAs in pork belly (*p* < 0.05). Although Liu Dengyong et al. [26] found no significant change in the SFA content of pork belly after braising, they observed an increasing trend in the monounsaturated fatty acid (MUFA) content and a decreasing trend in the polyunsaturated fatty acid (PUFA) content. Dietary fatty acid composition influenced fat deposition in rodents, chickens and pigs [27]. This study shows that the consumption of UFAs, MUFAs and PUFAs, and especially EPA and DHA, promoted glucose and lipid metabolism, as well as metabolic inflammation, gut microbiota and hepatic metabolism [28]. In this experiment, PUFAs in BCM pork fat showed the same trend as that in the research results of Gu et al. [25]. Of the three cooking methods, DCM yielded the highest ratio of UFAs/SFAs in pork fat, followed by BCM and SCM.

### 3.2. Effects of Consuming Pork Fat Cooked Using Different Methods on the Growth of Rats

**Figure 1:**
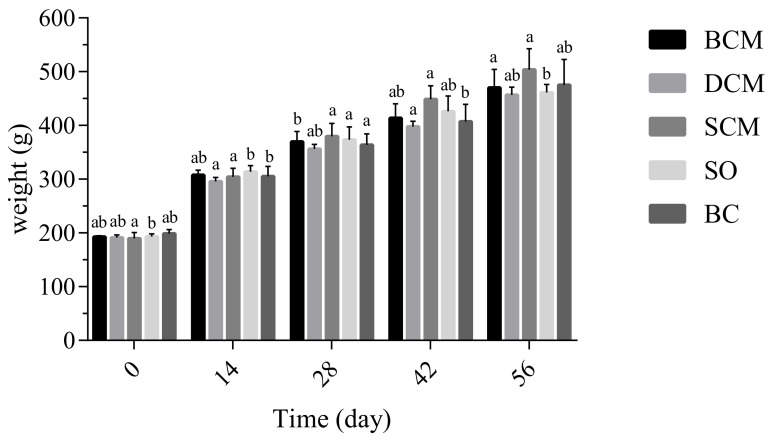
Effects of dietary fat cooked using different methods on the body weights of rats. Different letters at the same time point indicate significant differences (*p* < 0.05). BCM, braised cooking method; SCM, stewed cooking method; DCM, deep frying cooking method; SO, soybean oil; BC, blank controls.

As shown in Figure 1, all rats exhibited normal growth and similar body weights under normal feeding conditions. The body weight increased significantly when the BCM and SO groups were fed to 28 days; the increase in the DCM and SCM groups was not significant (*p* < 0.05). Comparing rats in different experimental groups, there was no significant difference in body weight between the groups. Liang et al. [29] found that HFD significantly increased body weight and visceral fat accumulation. Qiu Bofang et al. [30] studied the effects of different fatty acid compositions on body weight in rats and found no significant difference in this parameter throughout the experimental period. This is consistent with the experimental results of this study, indicating the feeding time at which there is no effect on the body weight of rats in the experimental groups corresponding to the three cooking methods.

### 3.3. Effects of Consuming Pork Fat Cooked Using Different Methods on Glucose and Lipid Metabolism in Rats

#### 3.3.1. Effects of Pork Fat Cooked Using Different Methods on Blood Glucose

**Figure 2:**
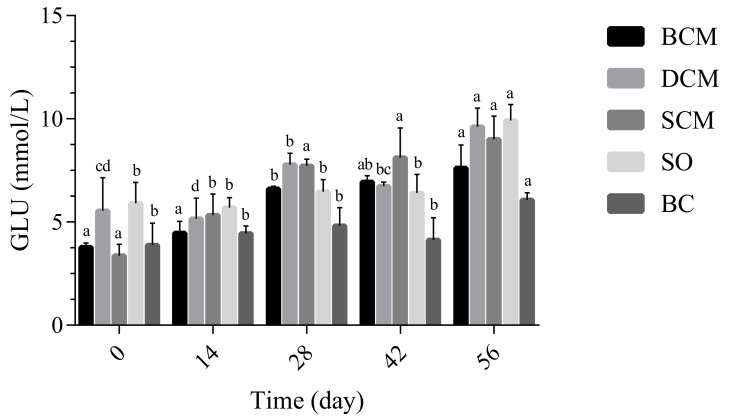
Effect of dietary fat cooked using different methods on glucose (GLU) levels in rats. Different letters at the same time point indicate significant differences (*p* < 0.05). BCM, braised cooking method; SCM, stewed cooking method; DCM, deep frying cooking method; SO, soybean oil; BC, blank controls.

Glycolipid metabolism is essential to life. As shown in Figure 2, as the experimental feeding time progressed, the level of GLU metabolism of rats in the BCM, SCM and DCM groups showed slowly increasing trends that differed significantly from the trend in the BC group. From the graph, the GLU level of rats in the BCM group showed no difference after 28 days of feeding. A significant difference occurred at 42 days of feeding. The DCM group showed an upward trend from 0 to 28 days and showed a decrease after feeding at 42 days. At 56 days, this group showed an upward trend again. There was no significant difference in GLU of rats in the SCM and SO groups during the feeding process.

In the 14 days in feeding, no significant differences in GLU levels were observed between the three cooking method groups. From day 28, however, the GLU levels of rats in the three cooking method groups began to differ significantly, such that the levels were significantly different in the BCM and DCM groups. The GLU levels of rats were significantly higher in the BCM and DCM groups. Liang et al. [29] studied the changes in blood glucose of mice during a test between a normal diet, high-fat diet and high-cholesterol diet and found no significant difference. However, a conflicting result was also found in mice fed with HFD (60% of the diet energy), showing both overweight and dyslipidemia in the mice [31]. De Araujo et al. [32] reported that animals fed a high-fat diet had a significant increase in blood GLU compared with animals fed a normal diet. We found similar trends in GLU levels in the BCM and DCM groups. The above results indicate that, compared with other experimental groups, pork fat subjected to DCM and SCM had a greater impact on GLU levels in rats.

#### 3.3.2. Effects of Pork Fat Cooked Using Different Methods on Serum Lipid Concentrations in Rats

As shown in Table 3, the serum TG levels of rats in the different cooking method groups increased slowly over the feeding period compared with the BC group. After day 42 of the experimental feeding period, the TG values increased significantly (*p* < 0.05), indicating that consumption of pork fat cooked using any of the three cooking methods led to increases in serum TG levels in rats. Compared with the BC group, the BCM and DCM groups showed significant increased TC levels on the 42nd and 56th days (*p* < 0.05), whereas similar changes were not observed in the SCM group. Compared with the values on day 0, the rats in the three cooking method groups exhibited slowly decreasing HDL-C values over the experimental period, with slow increases in the BCM and DCM groups after day 42. The HDL-C levels in the SCM experimental group decreased significantly by day 56. The HDL-C values of the rats in the SO and BC groups did not change significantly. With the extension of the feeding time, the HDL-C levels of rats in the BCM and DCM groups decreased significantly (*p* < 0.05), while the changes in the SCM group were not significant. Compared with the BC and SO groups, the LDL-C levels of the rats in all three cooking method groups gradually increased across the feeding period. This indicates that the consumption of pork fat cooked via different methods caused an increase in the LDL level of rats.

Yin Caina et al. [33] studied the intake of SFA-rich refined lard in a mouse model and observed associations of excessive intake with increases in serum TC, TG and LDL-C levels and a decrease in serum HDL-C levels. Zou Wenli et al. [34] studied the effects of different diets on serum lipid levels in rats but found no significant effects during a short period of time. The braised pork fat that had been stewed for a long time was shown to lower blood lipid levels [35]. Serum cholesterol levels often increase with age. An increase in the serum TC level leads to an increase in the LDL-C level, which can cause diseases such as atherosclerosis at very high levels [36] and thus is not conducive to human health. The content of LDL-C is related to the incidence and severity of cardiovascular disease [37]. Dietary SFA and cholesterol can increase the serum TC level. In our study, pork fat cooked using BCM had a lower SFA content than pork fat cooked using either SCM or DCM (Table 1) and thus is more suited to the health needs of consumers.

### 3.4. Effects of Pork Fat Cooked Using Different Methods on Liver Fat Deposition in Rats

**Figure 3:**
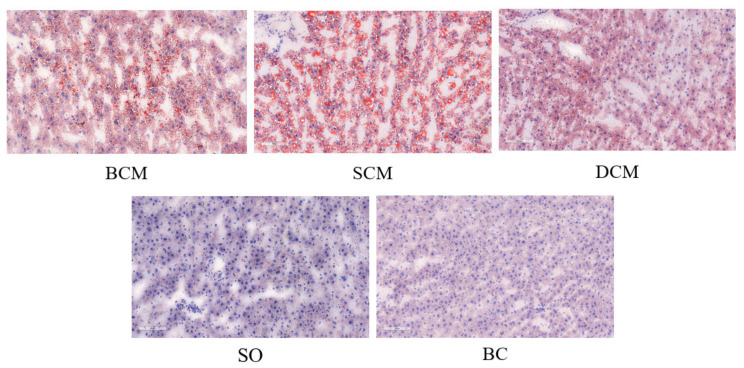
Oil red O staining of liver sections from rats fed with pork fat cooked using different methods. Note: Lipid droplets in the tissues are marked in red. Cell nuclei are shown in blue. The field of view is 100 µm. BCM, braised cooking method; SCM, stewed cooking method; DCM, deep frying cooking method; SO, soybean oil; BC, blank controls.

Figure 3 shows the liver fat deposition in rats that consumed pork fat cooked using the three different methods. Microscopical analysis of frozen liver tissue sections stained with oil red O revealed that the samples from the BCM and SCM groups had obvious red-stained areas. Abundant diffuse fat droplets of varying sizes were observed in the liver tissue of the BCM and SCM groups, with many large vesicle-like fat droplets. A few small, red-stained fat droplets were observed in the liver tissues of rats in the DCM group. Liver tissues from rats in the SO and BC groups contained the fewest fat droplets, and they were not easy to distinguish without a machine. Compared with the SO and BC groups, the BCM, SCM and DCM groups all showed different degrees of increase, with a darker color, a gradually expanded area of fat droplets, and the fat droplets distributed in different patterns. A higher amount of fat deposition was observed in the liver tissues of rats in the SCM group than in the BCM and DCM groups, and the difference was significant (*p* < 0.05).

**Figure 4:**
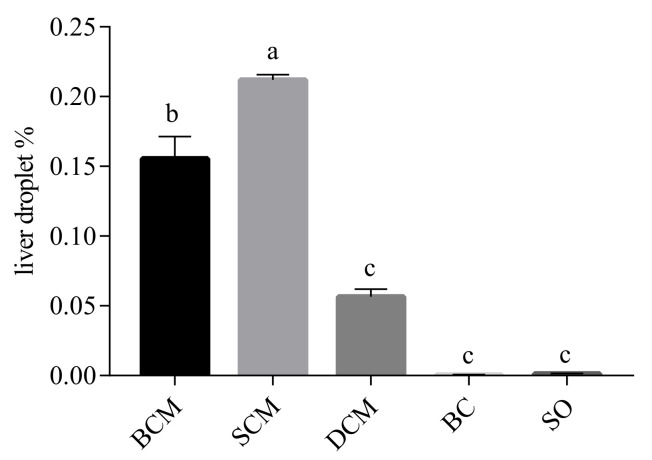
Effects of pork fat cooked using different methods on the liver droplet contents in rat liver tissues. Note: Different letters (a, b and c) indicate significant differences (*p* < 0.05). BCM, braised cooking method; SCM, stewed cooking method; DCM, deep frying cooking method; SO, soybean oil; BC, blank controls.

The liver fat deposition scores of rats in each experimental group were basically consistent with the observations from the stained tissue sections. As shown in Figure 4, the liver fat deposition index of rats in each experimental group was calculated by the NM algorithm. The fat drop ratio results show that the SCM group had the highest level of fat deposition in the liver, followed by the BCM group, DCM group, SO group and BC group, in order. The results show that compared with foods cooked using DCM, the consumption of foods cooked using SCM and BCM more significantly affected fat deposition in the livers of rats.

More than a quarter of adults in Western countries have symptoms of fat deposition in the liver, and this is the main cause of cardiovascular metabolic disorders and diabetes [38]. Charlotte et al. [39] found that mechanistically, dietary fat consumption may affect liver fat deposition because disordered phospholipid metabolism enables SFAs to upregulate the expression of genes involved in fat synthesis to promote fat deposition; furthermore, high concentrations of UFAs induce adipose cell apoptosis and reduce adipogenesis [40]. Cholesterol can also promote fat deposition in the liver. Chen Mengjie et al. [41] studied the induction of obesity in rats by intervention with a high-fat diet with different lipid components and observed a significantly higher level of fat deposition in the liver in rats fed lard than in rats fed with soybean oil. The significant reduction in liver fat in rats can be attributed to high n-3 PUFAs, and the reduced fat deposition in the livers of rats from the SO group may be due to the low SFA content and lack of TC in soybean oil [35]. Animal studies have shown that consuming C16:1 can regulate the liver fat deposition index [42]. We found that pork fat cooked using SCM or BCM had a higher C16:1 content than pork fat cooked using DCM. This may be the main reason that the liver fat deposition of rats in the DCM group was less than that in the BCM and SCM groups.

### 3.5. Effects of Pork Fat Subjected to Different Cooking Methods on Inflammatory Factors in the Liver Tissues of Rats

**Figure 5:**
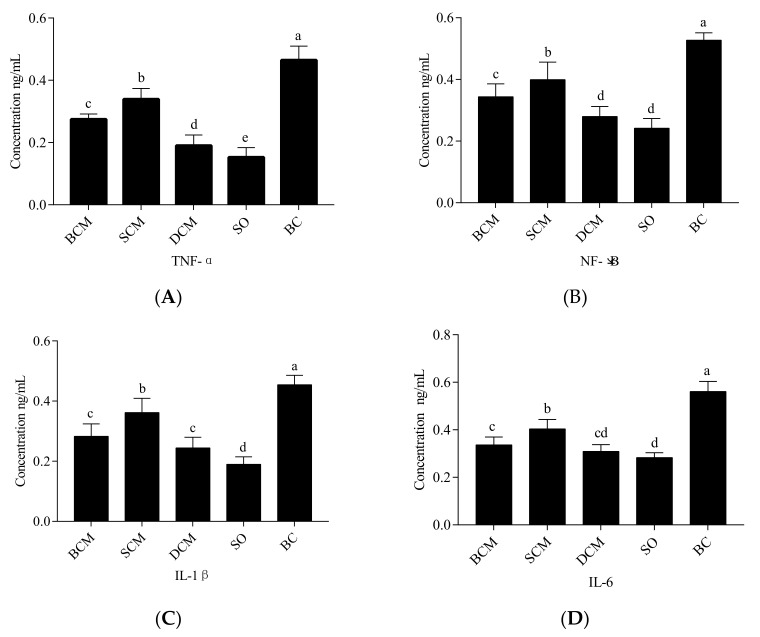
Effects of pork fat cooked using different methods on the concentrations of TNF-α (**A**), NF-κB (**B**), IL-β (**C**), and IL-6 (**D**) in rat liver tissues. Note: Different letters (a, b and c) indicate significant differences (*p* < 0.05). BCM, braised cooking method; SCM, stewed cooking method; DCM, deep frying cooking method; SO, soybean oil; BC, blank controls.

The concentrations of the inflammatory factors NF-κB, IL-β, IL-6 and TNF-α in the livers of rats that consumed pork fat cooked using different methods were measured by ELISAs. The results are shown in Figure 5. Compared with the BC group, the concentrations of NF-κB, IL-β, IL-6 and TNF-α in the livers of rats in the BCM, SCM and DCM groups were decreased (*p* < 0.05), indicating that the three cooking methods inhibited the inflammatory factor expression level in the liver. The significant differences in liver inflammation between rats in the SCM, BCM and DCM groups and those in the SO and BC groups indicate that the cooking method plays a role in the immunomodulatory effect of consumed pork fat in our rat model.

Significantly higher concentrations of NF-κB and TNF-α were observed in rats in the SCM group than in rats in the BCM and DCM groups, and the levels of inflammation in the three cooking method experimental groups were significantly different (*p* < 0.05). However, the concentrations of IL-β and IL-6 were not significantly different between the BCM and DCM groups (*p* > 0.05). The results suggest that, compared with the BC experimental group, the pork fat of the three cooking methods did not cause an inflammatory immune response. These changes in the expression of inflammatory factors in our experiments indicate that fat deposition and oxidative stress are closely related to inflammation. Dietary habits have been closely linked to inflammatory diseases [43]. NF-κB is a key transcription factor associated with inflammatory cytokines, such as many interleukins and chemokines. The transcriptional regulators of these genes are cytokines induced by NF-κB, such as TNF-α, IL-1β and IL-6; they are also potential activators of NF-κB [44], which generate automatic circulation, which is activated by ROS and inflammation, and can effectively destroy tissue parenchyma and damage liver function [45]. IL-6 has been reported as an important marker of the inflammatory response [46].

The lower concentrations of NF-κB in the BCM and DCM groups than in the SCM group may have been due to the lower SFA levels in pork fat cooked using either of the first two cooking methods. SFA was shown to activate NF-κB via Toll-like receptor 4 [47]. Schmitz et al. [48] revealed that n-3 and n-6 fatty acids act as ligands and modulators of NF-κB, which controls the expression of various genes encoding inflammatory factors and lipid metabolism components [49]. Accordingly, the concentrations of inflammatory factors were lower in the BCM, SCM and DCM groups than in the BC group. The fatty acid C16:1 can inhibit or delay inflammation in the body [42]. Excessive intake of SFAs accelerates immune responses and leads to the occurrence of chronic inflammation [50]. Saturated fatty acids, particularly palmitate, induce insulin resistance by activation of inflammatory signaling in cells [51]. IL-6, SOCS3 and TNF-α are associated with the induction of hepatic insulin resistance [52,53].

Pork fat cooked using SCM had a significantly higher level of C16:1 than pork fat cooked using BCM or DCM. The levels of SFAs and C18:2 were lower in pork fat cooked using BCM than in pork fat cooked using SCM or DCM. This may explain why lower levels of inflammation were observed in the BCM or DCM group than in the SCM group in our experiment.

### 3.6. Effects of Pork Fat Cooked Using Different Methods on the Rat Gut Microbiota Structure and Abundance of Bacteria

**Figure 6:**
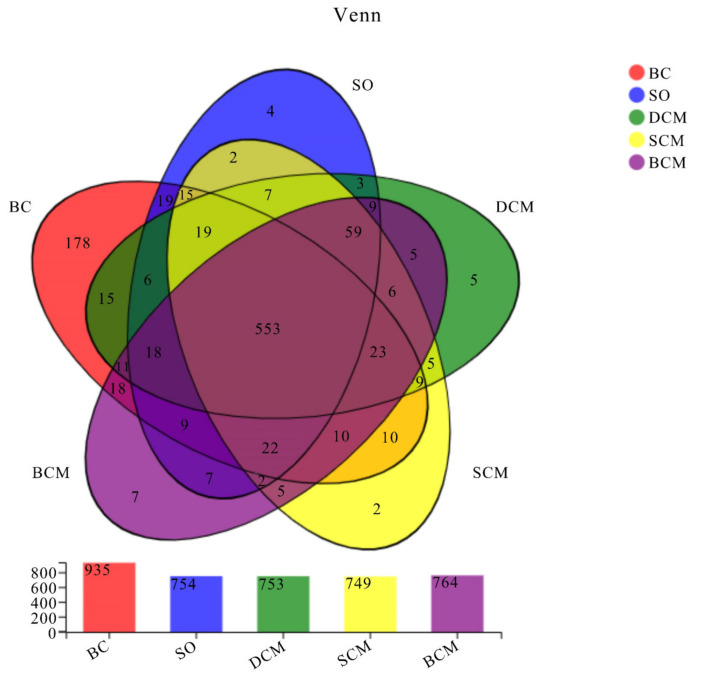
Venn diagram of operational taxonomic units in the gut microbiota of rats fed pork fat cooked using different methods. BCM, braised cooking method; SCM, stewed cooking method; DCM, deep frying cooking method; SO, soybean oil; BC, blank controls.

The gut microbiota of rats in all five groups was analyzed. The operational taxonomic unit (OTU) distribution diagrams are shown in Figure 6. Overall, 553 OTUs were shared by all experimental groups, while 7, 2, 5, 4 and 178 OTUs were unique to the BCM, SCM, DCM, SO and BC groups, respectively. Significant differences in the numbers of OTUs between the different groups of rats fed with pork fat cooked using different methods reflect the differences in the composition of the intestinal microbial flora between the different experimental groups due to differences in the cooking methods.

**Figure 7:**
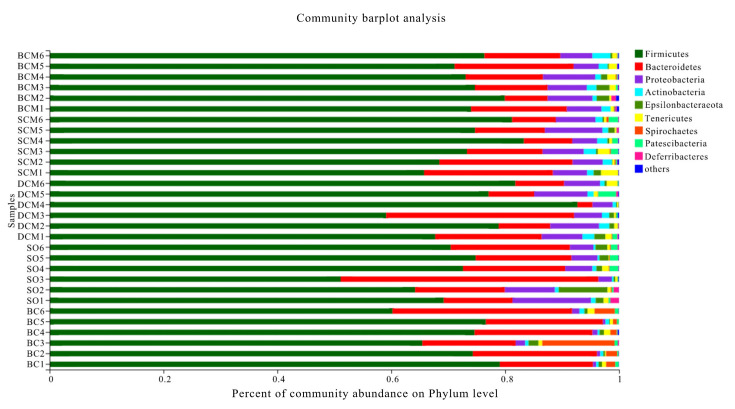
Phylum-level microbial composition in the gut microbiota of rats fed with pork fat cooked using different methods. BCM, braised cooking method; SCM, stewed cooking method; DCM, deep frying cooking method; SO, soybean oil; BC, blank controls.

As shown in Figure 7, at the phylum level, the distribution patterns differed between the experimental groups. Firmicutes, Proteobacteria and Bacteroidetes were the dominant bacterial phyla in the intestines of rats in all groups, except for Proteobacteria in the BC group. The abundances of all three phyla changed in the three cooking method groups relative to the SO or BC group (*p* < 0.05). Furthermore, a comparison with the SO group revealed a significant increase in the abundance of Firmicutes and a significant decrease in the abundance of Bacteroidetes in the BCM, SCM and DCM groups (Table 4). Compared with the BC group, the BCM, SCM, DCM and SO groups had a significantly increased abundance of Proteobacteria (*p* < 0.05). However, there were no significant differences between the BCM, SCM and DCM groups in terms of abundance at the phylum level.

Firmicutes and Bacteroidetes species in the gut have been closely linked to the health of the host [54]. Firmicutes and Bacteroidetes are the most predominant bacterial phyla in the rat intestine, where they affect the efficiency of energy extraction and are associated with obesity. The gut microbiota of obese animals was found to have a relatively high abundance of Firmicutes [55]. High-fat diet-induced obesity is associated with increases in the proportions of Firmicutes and Bacteroidetes [56], with effects on the overall metabolic functions of intestinal microbes. Lam et al. [57] studied a high-fat diet intervention in rats and found an increase in the abundance of Firmicutes and a decrease in the abundance of Bacteroidetes in the high-fat group relative to the control group, consistent with the results of this article. From the changes in the abundance of Firmicutes and Bacteroides, pork fat from the three cooking methods has a certain potential to promote the production of body obesity.

**Figure 8:**
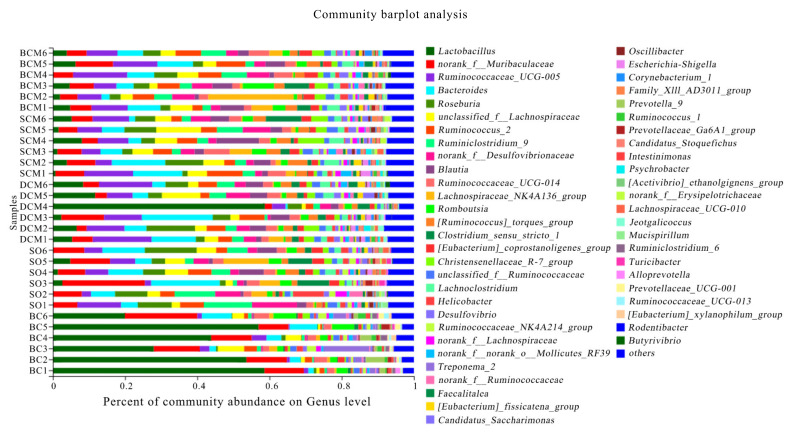
Microbial composition at the genus classification level in rats fed with pork fat cooked using different methods. BCM, braised cooking method; SCM, stewed cooking method; DCM, deep frying cooking method; SO, soybean oil; BC, blank controls.

Figure 8 shows the results of a statistical annotation analysis of microorganisms present in the gut microbiota of rats at proportions exceeding 1.00%. The analysis identified the following dominant genera: *Ruminococcaceae_UCG-005*, *Bacteroides*, *Lactobacillus*, *Roseburia, Blautia*, *unclassified_f__Lachnospiracea*, *norank_f__Muribaculaceae*, *Lachnospiraceae_NK4A136_group*, *Clostridium_sensu_stricto_1*, *Romboutsia*, *Lachnoclostridium*, *[Ruminococcus]_torques_group* and *Ruminococcus_2.* The experimental results reveal significant changes in most genera in response to the consumption of pork fat cooked using different methods (*p* < 0.05).

The genus *Ruminococcaceae* accounted for the largest proportion of the gut microbiota, with values of 9.31 ± 1.26% in the BCM group, 7.67 ± 1.07% in the SCM group, 10.74 ± 1.57% in the DCM group, 6.52 ± 2.02% in the SO group and 2.24 ± 0.75% in the BC group. The differences between the BC group and the other groups were significant.

In the gut microbiota of rats fed with the three types of pork fat cooked using different methods, the proportion of Bacteroides was highest in the SO group at 8.16 ± 1.58%, followed by the BCM group at 7.70 ± 1.14%, the DCM group at 7.27 ± 1.95%, the SCM group at 3.34 ± 2.24% and the BC group at 2.62 ± 0.60%, and the differences between the BC and SCM groups were significant (*p* < 0.05). Regarding the genus Blautia, the proportions did not differ between the three cooking method groups that were fed with pork fat cooked using different methods (*p* > 0.05), but the differences between the BC group and the other groups were significant (*p* < 0.05).

The proportion of the genus Lactobacillus also differed between the groups of rats, with the highest proportion in the BC group at 46.24 ± 6.75%, followed by 4.92 ± 0.50%, 2.40 ± 0.57%, 7.14 ± 2.18% and 0.42 ± 0.33% in the BCM, SCM, DCM and SO groups (Table 5), respectively. The differences between the BC experimental group and all of the other groups were significant (*p* < 0.05). The direction of changes in the proportion of the genus Roseburia was opposite to that in the proportion of Lactobacillus. In the BC group, Roseburia species accounted for only 0.64 ± 0.22% of the gut microbiota, compared with 9.73 ± 1.73% in the SO group (the highest proportion), 3.70 ± 0.55% in the BCM group, 4.39 ± 1.09% in the SCM group and 4.98 ± 0.92% in the DCM group.

The phylum- and genus-level classifications indicate that, in rats, consuming pork fat cooked using different methods affects the structure and function of the gut microbiota. Compared with the BC group and SO group, significant differences were observed in the gut microbiota of rats in the three cooking method groups. In humans, the human intestinal microflora is largely composed of species from the phyla Bacteroidetes and Firmicutes, followed by Actinomyces, Proteobacteria and Verrucomicrons [58]. Zou Fang et al. [59] found that different types of fatty acids have different effects on the physiological function, digestive function and gut microbiota of rats. In another study, diets containing fat from different sources had different effects on the cecal microflora of piglets [60]. Sefcikova et al. [61] found that diets with different fat contents can regulate the functional structure of intestinal microbes in rats before and after weaning. Compared with a low-fat and high-carbohydrate diet, a high-fat and low-carbohydrate diet was shown to significantly reduce the concentrations of SFCAs and the number of bifidobacteria in feces of male rats [62]. Zhang et al. [17] showed that, in rats, a high-fat diet intervention led to increases in the abundances of the phyla Firmicutes and Verrucobacteria, and the genus *Lactococcus*, and a decrease in the abundance of Proteobacteria, relative to the control group. Other studies have shown that long-term consumption of high-fat and SFA-rich diets had significant effects on the gut microbiota [63], particularly diversity and structural function, leading to dysfunction and disease [64]. Related studies have also focused on the correlation between the gut microbiota and obesity induced by a high-fat diet. For example, one study conducted a genetic analysis of the gut microbiota of obese mice and normal mice and found differences between the two groups. Specifically, the microbes in the intestines of obese mice were enriched for genes associated with energy metabolism, which enabled the host to more easily obtain energy from food and gain weight [65]. Another study showed that the gut microbiota was significantly related to susceptibility to obesity, insulin resistance, type 2 diabetes mellitus and other diseases [66]. Kübeck et al. [67] highlighted that the functional signals generated from interactions between intestinal microbiota and dietary components played an important role in host energy homeostasis and the development of obesity.

In the current study, which explored the relationship between dietary fat and intestinal microbes, the types and content of dietary fat, especially the effects of a high-fat diet on the whole body or gut microbiota of rats, were the main focus. The levels of *Bacteroides* and *Lactobacillus* in the rat intestine were downregulated, which was related to the SFA content of pork fat in the three cooking methods. Many *Lactobacillus* species are considered probiotics; these have been shown to not only reduce inflammation and mucosal damage in some inflammatory bowel disease models but also improve the overall physiology and lipid metabolism of the host [68]. *Lactobacillus* species can produce bile acid-binding hydrolase, which can hydrolyze bound bile acids to free acids. These free bile acids improve the enterohepatic circulation of cholesterol and promote its conversion to bile acid, thereby reducing the systemic cholesterol level [69]. This is consistent with our observation of the highest liver fat deposition index in the SCM group. This study only paid attention to changes in the gut microbiota of rats at the end of the experimental feeding period. However, the gut microbiota may have changed gradually over time. As the structure of the gut microbiota is dynamic, further research should focus on these dynamic changes in and regulation of flora across the experimental feeding period and the underlying mechanism of action.

## 4. Conclusions

This study explored the effects of pork fat cooked using different methods on indicators of growth, glucose and lipid metabolism, liver inflammation and gut microbiota health in rats. Pork fat cooked using BCM and SO led to significant increases in the body weights of rats. Compared to the BC group, pork fat cooked using DCM and SCM led to significant increases in GLU levels. Analysis of rat liver sections revealed the highest liver fat deposition index in the SCM group relative to the BCM and DCM groups. Compared with the BC group, rats in the BCM, SCM and DCM groups did not exhibit upregulated NF-κB expression in their livers, and the NF-κB expression levels in the SCM and BCM experimental groups were significantly reduced. There were significant differences in the gut microbiota between rats in the three pork fat groups, the BC group and the SO group. Specifically, a significantly lower abundance of Firmicutes was observed in the intestines of rats in the SO group relative to the three pork fat groups. The intestinal abundance of Proteobacteria was significantly lower in the BC group than in the other groups, and the abundance of Bacteroidetes was significantly lower in the BCM, SCM and DCM groups than in the BC and SO groups. Firmicutes and Bacteroidetes are the most predominant bacterial phyla in the body. The observed changes in the abundance of Firmicutes suggest that pork fat can promote obesity development, regardless of the cooking method. It is unbeneficial to intestinal health. In conclusion, this study reveals the nutritional value of pork fat cooked using different methods from the perspective of rat physiology and the gut microbiota and provides a theoretical basis for selecting healthy cooking methods and a reference for further research on its influence mechanism.

## Figures and Tables

**Table 1:** Formula and calculated nutrient composition of experimental diets.

Content	BCM	SCM	DCM	SO	BC
Casein	200.0	200.0	200.0	200.0	180.3
Corn starch	397.4	397.4	397.4	397.4	519.8
Dextrin	132.0	132.0	132.0	132.0	119.0
Sucrose	100.0	100.0	100.0	100.0	90.1
Pork fat of the BCM	70.0	-	-	-	-
Pork fat of the SCM	-	70.0	-	-	-
Pork fat of the DCM	-	-	70.0	-	-
Soybean oil	-	-	-	70.0	-
Crude fat					40.0
Cellulose	50.0	50.0	50.0	50.0	45.0
Minerals	35.0	35.0	35.0	35.0	31.5
Vitamins	10.0	10.0	10.0	10.0	9.0
L-cystine	3.0	3.0	3.0	3.0	2.7
Choline chloride	2.5	2.5	2.5	2.5	2.2
Tert-butyl hydrogen	0.01	0.01	0.01	0.01	0.01
Total (g)	1000	1000	1000	1000	1000
Total energy (kcal/g)	3.7	3.7	3.7	3.7	3.7
Protein (%)	19.4	19.4	19.4	19.4	19.4
Carbohydrate (%)	63.6	63.6	63.6	63.6	63.6
Fat (%)	16.9	16.9	16.9	16.9	9.67

BCM, braised cooking method; SCM, stewed cooking method; DCM, deep frying cooking method; SO, soybean oil; BC, blank controls.

**Table 2:** Fatty acid compositions of pork fat samples cooked using the three methods.

Fatty Acid	BCM	SCM	DCM	SO
C10:0	0.16 ± 0.03 ^a^	0.15 ± 0.03 ^a^	0.21 ± 0.03 ^a^	—
C12:0	0.07 ± 0.02 ^b^	0.26 ± 0.04 ^a^	0.16 ± 0.02 ^ab^	—
C16:0	69.05 ± 1.38 ^c^	133.70 ± 0.95 ^a^	103.24 ± 0.35 ^b^	11.04 ± 0.15 ^d^
C17:0	0.64 ± 0.14 ^b^	2.82 ± 0.26 ^a^	1.10 ± 0.05 ^b^	0.05 ± 0.005 ^c^
C18:0	38.33 ± 4.72 ^c^	91.64 ± 2.08 ^a^	54.67 ± 0.25 ^b^	4.67 ± 0.18 ^d^
C20:0	0.85 ± 0.15 ^b^	2.18 ± 0.23 ^a^	1.59 ± 0.01 ^a^	0.40 ± 0.005 ^c^
C14:1	4.03 ± 0.81 ^b^	12.82 ± 1.05 ^a^	7.41 ± 0.49 ^b^	0.01 ± 0.00 ^d^
C16:1	5.35 ± 1.07 ^b^	21.15 ± 1.58 ^a^	17.44 ± 0.17 ^a^	2.12 ± 0.01 ^b^
C17:1	0.35 ± 0.08 ^c^	1.68 ± 0.12 ^a^	0.87 ± 0.01 ^b^	0.03 ± 0.00 ^d^
C18:1	94.66 ± 1.93 ^b^	174.39 ± 1.09 ^a^	151.29 ± 10.75 ^a^	22.21 ± 0.23 ^d^
C20:1	3.27 ± 0.50 ^b^	8.84 ± 0.75 ^a^	4.64 ± 0.02 ^b^	0.18 ± 0.01 ^c^
C18:2	58.27 ± 2.81 ^b^	103.85 ± 1.07 ^a^	96.48 ± 6.72 ^a^	50.77 ± 1.83 ^b^
C20:2	2.98 ± 0.48 ^b^	7.82 ± 0.68 ^a^	3.91 ± 0.00 ^b^	—
C18:3	0.58 ± 0.14 ^a^	1.37 ± 0.14 ^a^	0.52 ± 0.39 ^a^	6.11 ± 0.23 ^b^
C20:4	1.30 ± 0.23 ^c^	3.31 ± 0.30 ^a^	2.30 ± 0.04 ^b^	—
C22:4	0.08 ± 0.01 ^b^	0.55 ± 0.05 ^a^	0.18 ± 0.01 ^b^	—
C20:5	0.25 ± 0.00 ^b^	0.52 ± 0.08 ^a^	0.50 ± 0.03 ^a^	—
SFAs	109.09 ± 6.45 ^c^	222.74 ± 3.59 ^a^	160.97 ± 0.54 ^b^	16.63 ± 0.35 ^d^
MUFAs	107.66 ± 4.40 ^c^	218.87 ± 4.61 ^a^	181.66 ± 10.40 ^b^	24.54 ± 0.21 ^d^
PUFAs	63.46 ± 3.67 ^b^	117.43 ± 2.33 ^a^	103.90 ± 7.13 ^a^	57.91 ± 1.05 ^c^
UFAs	171.12 ± 8.08 ^b^	336.30 ± 6.93 ^a^	285.56 ± 17.53 ^a^	81.45 ± 2.27 ^d^
UFAs/SFAs	1.57 ± 0.03 ^b^	1.51 ± 0.01 ^b^	1.77 ± 0.10 ^a^	5.35 ± 0.21 ^a^

Different letters in the same row indicate significant differences (*p* < 0.05). BCM, braised cooking method; SCM, stewed cooking method; DCM, deep frying cooking method; SO, soybean oil; BC, blank controls.

**Table 3:** Effects of pork fat cooked using different methods on serum lipid concentrations in rats over time.

Lipid	Day	BCM	DCM	SCM	SO	BC
TG (mmol/L)	0	0.74 ± 0.16 ^d^	0.84 ± 0.15 ^c^	0.53 ± 0.09 ^d^	0.68 ± 0.11 ^b^	0.81 ± 0.12 ^d^
	14	1.37 ± 0.16 ^bc^	1.38 ± 0.31 ^b^	1.23 ± 0.14 ^bc^	1.36 ± 0.14 ^a^	1.19 ± 0.24 ^ab^
	28	1.07 ± 0.22 ^c^	0.86 ± 0.32 ^c^	0.86 ± 0.22 ^cd^	1.12 ± 0.14 ^ab^	0.87 ± 0.14 ^bc^
	42	1.65 ± 0.11 ^b^	1.21 ± 0.33 ^bc^	1.60 ± 0.29 ^b^	1.72 ± 0.61 ^a^	1.14 ± 0.41 ^abc^
	56	2.26 ± 0.65 ^a^	2.22 ± 0.35 ^a^	2.2 ± 0.85 ^a^	1.74 ± 0.74 ^a^	1.35 ± 0.26 ^a^
TC (mmol/L)	0	2.36 ± 0.17 ^a^	2.42 ± 0.21 ^a^	2.32 ± 0.17 ^a^	2.08 ± 0.52 ^a^	1.63 ± 0.47 ^a^
	14	1.98 ± 0.14 ^b^	1.94 ± 0.39 ^bc^	1.90 ± 0.18 ^bc^	2.15 ± 0.18 ^a^	1.84 ± 0.16 ^a^
	28	1.46 ± 0.13 ^c^	1.68 ± 0.48 ^c^	1.55 ± 0.10 ^d^	1.37 ± 0.12 ^b^	1.50 ± 0.16 ^a^
	42	1.85 ± 0.14 ^b^	1.81 ± 0.25 ^c^	1.72 ± 0.16 ^cd^	1.64 ± 0.20 ^b^	1.47 ± 0.12 ^a^
	56	2.22 ± 0.23 ^a^	2.33 ± 0.20 ^ab^	1.96 ± 0.13 ^b^	2.12 ± 0.22 ^a^	1.52 ± 0.21 ^a^
HDL-C (mmol/L)	0	0.94 ± 0.02 ^a^	0.97 ± 0.05 ^a^	0.94 ± 0.10 ^a^	0.92 ± 0.08 ^a^	0.84 ± 0.06 ^a^
	14	0.70 ± 0.02 ^b^	0.73 ± 0.13 ^b^	0.76 ± 0.07 ^b^	0.72 ± 0.06 ^b^	0.69 ± 0.09 ^b^
	28	0.57 ± 0.12 ^c^	0.60 ± 0.06 ^c^	0.64 ± 0.06 ^c^	0.57 ± 0.04 ^c^	0.55 ± 0.07 ^c^
	42	0.60 ± 0.05 ^c^	0.61 ± 0.05 ^c^	0.62 ± 0.05 ^c^	0.56 ± 0.08 ^c^	0.52 ± 0.06 ^c^
	56	0.62 ± 0.06 ^bc^	0.66 ± 0.06 ^bc^	0.57 ± 0.06 ^c^	0.62 ± 0.03 ^c^	0.47 ± 0.04 ^c^
LDL-C (mmol/L)	0	0.43 ± 0.08 ^c^	0.45 ± 0.09 ^b^	0.37 ± 0.05 ^b^	0.42 ± 0.03 ^c^	0.44 ± 0.06 ^c^
	14	0.34 ± 0.09 ^d^	0.27 ± 0.06 ^c^	0.22 ± 0.01 ^c^	0.35 ± 0.05 ^d^	0.40 ± 0.02 ^c^
	28	0.77 ± 0.04 ^b^	0.84 ± 0.09 ^a^	0.80 ± 0.03 ^a^	0.76 ± 0.06 ^b^	0.89 ± 0.04 ^a^
	42	0.88 ± 0.02 ^a^	0.85 ± 0.03 ^a^	0.79 ± 0.02 ^a^	0.84 ± 0.06 ^a^	0.89 ± 0.09 ^a^
	56	0.88 ± 0.05 ^a^	0.89 ± 0.07 ^a^	0.79 ± 0.03 ^a^	0.86 ± 0.07 ^a^	0.78 ± 0.06 ^b^

Different letters in the same column indicate significant differences (*p* < 0.05). BCM, braised cooking method; SCM, stewed cooking method; DCM, deep frying cooking method; SO, soybean oil; BC, blank controls.

**Table 4:** Microbial composition at the phylum classification level in rats fed with pork fat cooked using different methods.

	BCM (%)	SCM (%)	DCM (%)	BC (%)	SO (%)
Firmicutes	74.72 ± 2.67 ^a^	74.40 ± 6.24 ^a^	76.14 ± 10.63 ^a^	71.75 ± 6.71 ^a^	66.84 ± 7.73 ^b^
Proteobacteria	6.71 ± 1.52 ^a^	6.68 ± 1.82 ^a^	6.64 ± 1.95 ^a^	0.92 ± 0.46 ^b^	6.36 ± 3.77 ^a^
Bacteroidetes	14.13 ± 4.10 ^b^	14.54 ± 6.16 ^b^	13.36 ± 9.98 ^b^	21.10 ± 5.14 ^a^	21.65 ± 10.87 ^a^
Deferribacteres	0.23 ± 0.27 ^ab^	0.13 ± 0.13 ^ab^	0.12 ± 0.13 ^ab^	0.05 ± 0.07 ^b^	0.47 ± 0.57 ^a^
Epsilonbacteraeota	1.03 ± 0.93 ^a^	0.57 ± 0.49 ^a^	0.70 ± 0.66 ^a^	0.68 ± 0.52 ^a^	2.49 ± 2.76 ^a^
Tenericutes	1.05 ± 0.34 ^a^	1.17 ± 1.10 ^a^	0.82 ± 0.56 ^a^	0.84 ± 0.39 ^a^	0.61 ± 0.34 ^a^
Actinobacteria	1.65 ± 0.75 ^a^	1.52 ± 0.39 ^a^	1.31 ± 0.50 ^a^	0.62 ± 0.23 ^b^	0.56 ± 0.20 ^b^
Patescibacteria	0.14 ± 0.11 ^a^	0.74 ± 0.65 ^a^	0.77 ± 1.10 ^a^	0.43 ± 0.20 ^a^	0.83 ± 0.59 ^a^
Spirochaetes	0.06 ± 0.07 ^b^	0.13 ± 0.15 ^b^	0.05 ± 0.05 ^b^	3.53 ± 4.07 ^a^	0.12 ± 0.06 ^b^

Note: Different letters in the same row indicate significant differences (*p* < 0.05). BCM, braised cooking method; SCM, stewed cooking method; DCM, deep frying cooking method; SO, soybean oil; BC, blank controls.

**Table 5:** Microbial composition at the genus level in rats fed with pork fat cooked using different methods.

	BCM (%)	SCM (%)	DCM (%)	BC (%)	SO (%)
*Ruminococcaceae*_*UCG-005*	9.31 ± 1.26 ^a^	7.67 ± 1.07 ^a^	10.74 ± 1.57 ^a^	2.24 ± 0.75 ^b^	6.52 ± 2.02 ^a^
*Bacteroides*	7.70 ± 1.14 ^a^	3.34 ± 2.24 ^b^	7.27 ± 1.95 ^a^	2.62 ± 0.60 ^b^	8.16 ± 1.58 ^a^
*Roseburia*	3.70 ± 0.55 ^bc^	4.39 ± 1.09 ^b^	4.98 ± 0.92 ^b^	0.64 ± 0.22 ^c^	9.73 ± 1.73 ^a^
*Lactobacillus*	4.92 ± 0.50 ^b^	2.40 ± 0.57 ^c^	7.14 ± 2.18 ^b^	46.24 ± 6.75 ^a^	0.42 ± 0.33 ^b^
*Lachnospiraceae_NK4A136_group*	2.66 ± 1.29 ^ab^	4.55 ± 0.60 ^a^	0.32 ± 0.03 ^b^	1.18 ± 0.38 ^b^	1.38 ± 0.98 ^b^
*Blautia*	5.15 ± 0.74 ^a^	2.36 ± 0.71 ^ab^	5.57 ± 2.10 ^a^	0.11 ± 0.05 ^b^	3.97 ± 1.38 ^a^
*Romboutsia*	4.24 ± 1.51 ^ab^	7.18 ± 1.78 ^a^	1.80 ± 0.29 ^b^	2.19 ± 0.31 ^b^	1.74 ± 0.50 ^b^
*Clostridium_sensu_stricto_1*	2.92 ± 1.49 ^b^	5.51 ± 0.49 ^a^	1.75 ± 0.37 ^bc^	0.36 ± 0.17 ^c^	1.19 ± 0.40 ^bc^
*Ruminococcus_2*	6.06 ± 1.17 ^a^	5.71 ± 2.12 ^a^	4.84 ± 0.17 ^ab^	1.78 ± 0.72 ^b^	5.06 ± 0.88 ^ab^
*Ruminiclostridium_9*	3.73 ± 1.08 ^b^	4.04 ± 1.61 ^b^	2.78 ± 0.12 ^b^	0.80 ± 0.15 ^b^	8.83 ± 2.04 ^a^
*Ruminococcaceae_UCG-014*	3.20 ± 0.85 ^a^	3.99 ± 1.06 ^a^	4.79 ± 1.95 ^a^	2.34 ± 0.43 ^a^	2.43 ± 0.27 ^a^
*norank_f__Muribaculaceae*	7.11 ± 1.11 ^b^	1.04 ± 0.29 ^d^	3.33 ± 0.93 ^c^	11.56 ± 0.42 ^a^	7.71 ± 0.41 ^b^
*unclassified_f__Lachnospiraceae*	4.03 ± 0.80 ^a^	2.08 ± 0.40 ^a^	8.42 ± 3.79 ^a^	3.33 ± 1.54 ^a^	4.41 ± 0.92 ^a^
*[Eubacterium]_coprostanoligenes_group*	2.48 ± 0.41 ^b^	1.08 ± 0.29 ^b^	1.88 ± 0.23 ^ab^	1.02 ± 0.29 ^ab^	1.77 ± 0.55 ^a^
*Christensenellaceae_R-7_group*	2.38 ± 0.67 ^c^	1.15 ± 0.27 ^c^	3.07 ± 0.23 ^c^	0.48 ± 0.08 ^b^	1.15 ± 0.31 ^b^
*norank_f__Desulfovibrionaceae*	3.67 ± 0.68 ^b^	0.74 ± 0.19 ^b^	3.56 ± 1.19 ^ab^	0.36 ± 0.11 ^ab^	6.14 ± 2.36 ^a^
*[Ruminococcus]_torques_group*	3.03 ± 0.31 ^b^	0.81 ± 0.41 ^b^	2.27 ± 0.35 ^a^	—	2.11 ± 0.62 ^a^
*Lachnoclostridium*	2.45 ± 0.52 ^c^	3.77 ± 1.13 ^bc^	1.30 ± 0.42 ^bc^	—	1.55 ± 0.52 ^a^
*Faecalitalea*	1.29 ± 0.35 ^d^	0.58 ± 0.11 ^c^	1.70 ± 0.20 ^bc^	—	0.73 ± 0.81 ^a^
*Desulfovibrio*	1.12 ± 0.42 ^b^	—	2.32 ± 0.65 ^b^	0.49 ± 0.21 ^b^	1.03 ± 0.18 ^a^
*unclassified_f__Ruminococcaceae*	1.53 ± 0.14 ^b^	4.83 ± 1.16 ^b^	1.24 ± 0.36 ^b^	1.41 ± 0.25 ^b^	1.86 ± 0.28 ^a^
*[Eubacterium]_fissicatena_group*	—	5.28 ± 2.28	0.79 ± 0.57	—	0.69 ± 0.30
*norank_f__Erysipelotrichaceae*	—	3.93 ± 2.32	0.24 ± 0.21	0.04 ± 0.01	0.17 ± 0.20
*norank_f__norank_o__Mollicutes_RF39*	—	3.61 ± 0.35	0.65 ± 0.18	0.64 ± 0.34	0.69 ± 0.27
*Escherichia-Shigella*	—	2.25 ± 0.70	1.05 ± 0.59	—	0.42 ± 0.51
*norank_f__Peptococcaceae*	—	1.96 ± 2.43	0.59 ± 0.19	0.06 ± 0.02	0.32 ± 0.20
*Candidatus_Saccharimonas*	—	0.42 ± 0.12	1.02 ± 0.60	0.39 ± 0.20	0.84 ± 0.54
*Helicobacter*	—	0.60 ± 0.30	0.51 ± 0.04	0.85 ± 0.55	3.26 ± 3.08
*norank_f__Ruminococcaceae*	—	0.63 ± 0.31	0.11 ± 0.19	0.36 ± 0.07	1.38 ± 1.33
*Ruminococcaceae_NK4A214_group*	—	—	0.80 ± 0.26	1.24 ± 0.73	1.39 ± 0.29
*norank_f__Lachnospiraceae*	—	0.69 ± 0.10	—	—	1.24 ± 1.14
*Treponema_2*	—	—	—	4.35 ± 4.84	0.14 ± 0.04
*Prevotella_9*	—	—	—	2.60 ± 1.74	—
*Ruminiclostridium_6*	—	—	—	1.00 ± 0.54	—

Note: Different letters (a, b and c) in the same row indicate significant differences (*p* < 0.05). BCM, braised cooking method; SCM, stewed cooking method; DCM, deep frying cooking method; SO, soybean oil; BC, blank controls.

## Data Availability

The datasets generated for this study are available from the authors.
